# Effect of Dietary Protein Sources Substituting Soybean Meal on Growth Performance and Meat Quality in Ducks

**DOI:** 10.3390/ani10010133

**Published:** 2020-01-14

**Authors:** Joanna Kuźniacka, Jakub Biesek, Mirosław Banaszak, Andrzej Rutkowski, Sebastian Kaczmarek, Marek Adamski, Marcin Hejdysz

**Affiliations:** 1 Department of Animal Breeding, Faculty of Animal Breeding and Biology, UTP-University of Science and Technology in Bydgoszcz, Mazowiecka 28, 85-084 Bydgoszcz, Poland; kuzniacka@utp.edu.pl (J.K.); jakub.biesek@utp.edu.pl (J.B.); miroslaw.banaszak@utp.edu.pl (M.B.); adamski@utp.edu.pl (M.A.); 2 Department of Animal Nutrition and Feed Management, UP Poznań University of Life Sciences, Wołyńska 33, 60-637 Poznań, Poland; marhej@o2.pl (A.R.); sebastian.kaczmarek@up.poznan.pl (S.K.)

**Keywords:** body weight, carcass, dressing percentage, duck, fat content, legume seeds, muscle content

## Abstract

**Simple Summary:**

The presented results are from a scientific trial, in which vegetable protein from legumes was tested as an alternative to soybean meal in feed for Pekin ducks. Meat traits were evaluated, and the results showed that yellow lupin and rapeseed meal can be used as a substitute for soybean meal, as they provide similar productivity effectiveness and meat quality traits. The results demonstrate that there is a possibility of presenting a wider choice for duck producers and the consumer market.

**Abstract:**

The study aimed to examine the growth performance and meat quality of Pekin ducks fed diets consisting of various protein source alternatives (groups: II—yellow lupin (YL) and rapeseed meal (RSM); III—YL and narrow-leaved lupin (NLL); IV—pea and YL; V—RSM, YL, NLL and pea) to (I) soybean meal (SBM) and RSM. Four hundred and twenty ducks were assigned to five groups with six replicates (14 birds per group). After 7 weeks, 10 ducks from each group were slaughtered. Breast muscles were analyzed for water-holding capacity, drip loss, color, and elasticity. Productivity parameters did not differ between groups I and II but were lower in V. The weight of carcass, neck with skin, skin with subcutaneous fat and total fat were highest in group II. The proportion of wings was higher in group V. In group II, lightness (L*) was higher, but redness (a*) was lower. In groups, I and III, L* was lower and a* was higher. Breast muscles contained more protein in groups I and II, more fat in groups I and III and more water in groups II and IV. The inclusion of vegetable protein alternatives to SBM in duck diets provided the best results in birds fed with YL and RSM (ratio of 1:0:31 in starter and 1:0.81 in grower).

## 1. Introduction

In terms of global poultry meat production, ducks constitute the third-largest poultry production sector [1]. Duck meat is characterized by a darker red color compared with chicken meat. It also has a high content of polyunsaturated fatty acids, which are beneficial for human health. Ducks are also reared for eggs and feathers [1,2,3,4,5]. Aside from genotype or living conditions, diet is a key factor affecting the quality of the produced meat. Controlling birds’ dietary protein content allows producers to obtain the right amount of good-quality meat (muscle tissue). Due to the high costs of feeding birds, it is very important to search for alternative dietary components. Soybean meal (SBM) is a popular dietary ingredient in poultry feed mixture formulations because of its high protein content. Studies on substitutes for soybean meal have mainly focused on the use of rapeseed meal (RSM) and legume seeds, including lupins and peas, which are protein-rich feed components. New cultivars of legume plants contain very low levels of antinutrients and have protein levels comparable to soybean meal [6,7,8]. The protein of lupins is used as a replacement for soybean meal protein. For the old cultivars of a pea, the main danger was the high level of tannins and trypsin inhibitors, which had an effect on lower nutrient digestibility and was the reason for the unpopularity of using legumes in poultry feed. However, plant breeders have succeeded in developing new legume varieties, for example, pea seeds characterized by high starch content and slower degradation in intestines compared with other plants used in poultry feed [9,10]. The study aimed to examine growth performance and meat quality of Pekin ducks fed diets including various protein sources as an alternative to SBM.

## 2. Materials and Methods

Meat quality research does not require the consent of an ethical committee, and the slaughter was carried out in a professional slaughterhouse. The experiment was treated as a routine production cycle in small-scale conditions. Our research aim was mainly to analyze the quality of carcasses and meat.

### 2.1. Legume Seeds

Yellow lupin (*Lupinus luteus* cv. Mister), narrow-leaved lupin (*Lupinus angustifolius* cv. Sonet), and pea seeds (*Pisum sativum* cv. Tarachalska) were obtained from plant breeding stations (Wiatrowo, Poland). Seeds were ground using a model RG11 hammer mill (Zuptor, Gostyń, Poland) with a screen size of 2.0 mm. The chemical compositions of the legume seeds are presented in Table 1.

### 2.2. Bird Management and Sample Collection

One-day-old English Pekin ducks (420 birds) kept in pens were randomly assigned to five groups with six replicates (14 birds in each: 7 male and 7 female). The environmental conditions complied with standard requirements for growing ducks. Ducks were reared for 49 days. The birds and feed refuse were weighed on day 1^s^ and the last day of every feeding phase in the experiment (days 1, 28, 29, and 49). Bodyweight gain (BWG), feed intake (FI), and feed conversion ratio (FCR) were calculated. The ducklings had ad libitum access to drinking water and complete feed mixtures (diets in mash form). At the end of the experiment, from each dietary treatment, 10 birds (5 male and 5 female) were selected and weighed, and after 12 h of starvation, they were slaughtered and eviscerated. Plucked and gutted carcasses were analyzed in a laboratory for quality parameters.

### 2.3. Experimental Diets

Over the course of 49 experimental days, the birds were fed feed mixtures—starter (days 1–28) and grower (days 29–49)—which were based on different protein concentrates (45% in complete diets) containing as the protein sources yellow and narrow-leaved lupin seeds, pea seeds, potato protein, and RSM. In the starter feeding period, the protein concentrates were mixed with 25% maize and 30% triticale; in the grower period, they were mixed only with 55% triticale. The chemical composition of the concentrates is presented in Table 2 and Table 3. The shares of particular components in the protein concentrates were defined to provide the optimal amino acid (AA) content by minimization of the levels of antinutritional factors. The complete diets were isonitrogenous and had crude protein contents of about 200 g/kg (starter) and 180 g/kg (grower) and metabolic energy of 11.9 MJ/kg (Table 2 and Table 3). The nutrient content of the diets was calculated according to the recommended allowances and nutritive value of feedstuffs [11].

### 2.4. Meat Quality

The pH value of breast muscles was measured 15 min postmortem (pH_15_). Carcasses were chilled at 2 °C for 24 h and pH was measured again (pH_24_). The test of pH was done at a depth of 2.5 cm below the surface of the breast muscles using an Elmetron CX-701 pH meter (Elmetron, Zabrze, Poland) with a knife electrode. Duck carcasses were weighed using RADWAG scales (Radwag, Radom, Poland) with accuracy to the nearest 0.01 g and dissected [12]. The following parts were separated: breast muscles, leg muscles, skin with subcutaneous fat, visceral fat, offal (liver, heart, and stomach), wings with skin, neck with skin, and carcass remains (body and leg bones). Each carcass part was weighed. The color of the breast muscles was assessed using a colorimeter (Konica Minolta, model CR400, Tokyo, Japan). The device was calibrated using the white calibration plate no. 21033065 and the D_65_Y_86.1_x_0.3188_y_0.3362_ scale. The color was graded according to the CIE Lab system for lightness (L*), redness (a*), and yellowness (b*) [13]. To analyze drip loss, breast muscles were weighed postmortem (M1), suspended in transparent bags with holes for water leakage, and after 24 h of storage at 2 °C, were weighed again (M2) [14]. The water-holding capacity of breast muscles was analyzed using a modified method proposed by Grau and Hamm [15]. For that purpose, pooled samples (about 0.300 g) of disintegrated muscles were wrapped in Whatman grade 1 filter paper and kept under 2 kg of pressure for 5 min. The water-holding capacity of the meat was calculated based on the difference in weight before and after the test. Strength parameters of femoral bones (i.e., maximum load capacity (N) and compressive strength at maximum load (Mpa)) were analyzed on an INSTRON 3345 column system (Instron, Buckinghamshire, UK). Bluehill Instron software 3.0, 3345, 2013 was used for this purpose, and the bone load was simulated with the BEND FIXTURE 10 mm ANVIL adapter. The elasticity of the breast muscles was tested with the same technique.

### 2.5. Analytical Methods

For chemical analyses, representative samples of seeds were ground to pass through a 0.5 mm sieve. Legume seeds and other components were analyzed in duplicate for crude protein (CP) and ether extract (EE) using methods 976.05 and 920.39, respectively, according to Association of Official Analytical Chemists (AOAC) procedures (2007). Additionally, acid detergent fiber (ADF), expressed inclusive of residual ash and neutral detergent fiber (NDF), with heat-stable amylase and expressed inclusive of residual ash, were analyzed in seeds (942.05 and 973.18, respectively, according to AOAC [16]). The starch content in pea seeds was determined using a diagnostic assay kit for agricultural industries (Megazyme International; AOAC, 2005: Method 996.11, Dublin, Ireland) based on the use of thermostable α-amylase and amyloglucosidase. The AA content was determined on an AAA-400 Automatic Amino Acid Analyzer (INGOS s.r.o., Prague, Czech Republic), using ninhydrin for postcolumn derivatization (procedure 994.12; AOAC [16]). In the pea sample, the tannin content was analyzed according to the method of Kuhla and Ebmeier [17]. The raffinose family oligosaccharides (RFO) were extracted and analyzed by high-resolution gas chromatography, as presented by Zalewski et al. [18]. Phytate was determined according to the method of Haug and Lantzsch [19]. Lupin alkaloids were extracted from flour by trichloroacetic acid and methylene chloride (Sigma-Aldrich, Munich, Germany). The determination of alkaloids was carried out via gas chromatography (GC) (Shimadzu GC17A, Kyoto, Japan) using a capillary column (Phenomenex, Torrance, CA, USA).

### 2.6. Statistical Evaluation

All data were calculated in a statistical program. Analyses were performed with the appropriate procedures (one-way ANOVA) using STATISTICA 10.0 software PL, 2011. Statistical analysis of the results was performed using the general linear model procedure (SAS Institute Inc., Cary, NC, USA, 2007). The significance of differences was verified by Scheffé’s test at the significance level *p*-value < 0.05. Values less than 0.05 were statistically significantly different. All data are presented as means with a pooled standard error of the mean.

## 3. Results

### 3.1. Chemical Composition of Legumes

Table 1 presents the chemical composition of the analyzed seeds of pea (cv. Tarachalska) and yellow (cv. Mister) and narrow-leaved (cv. Sonet) lupins. Yellow lupin seeds contained higher levels of CP (42.47%) than pea and narrow-leaved lupin. The lowest level of ADF and NDF was found in pea seeds. However, the CF level was highest in narrow-leaved lupin seeds. Starch was found only in pea seeds. The content of total alkaloids was highest in yellow lupin seeds. However, it was at a low level (0.00085 g/kg) compared with alkaloids in narrow-leaved lupin seeds (0.00017 g/kg). We found a lack of alkaloids in pea seeds; however, the opposite situation was noticed for tannin content. The highest levels of oligosaccharides and raffinose were found in yellow lupin seeds (134.27 and 10.21 g/kg, respectively). Phytic phosphorus (p-phyt.) content was highest in yellow lupin seeds (0.74%); its level was lower in narrow-leaved lupin seeds (0.48%), and the lowest level was found in pea seeds (0.24%).

### 3.2. Duck Performance

The health status of the ducks was very good, and the mortality cases were only incidental. During the entire rearing period, four deaths were recorded at the beginning of rearing (weak duck chicks). The analysis of growth performance is shown in Table 4. During the first stage of rearing (days 1–28), the statistically significantly highest BWG was found in the control group (I), whereas in group V, it was the lowest (*p* < 0.05). Similarly, statistical differences were found throughout the rearing period (days 1–49), where ducks from the first group were characterized by BWG of 3182 g and ducks from group V of 3046 g (*p* < 0.05). Statistically, the highest feed consumption per kilogram of weight gain (FCR) was found in group V (2.18 kg/kg) compared with group I (2.04 kg/kg) in the first period of the experiment (days 1–28). FCR analysis for the entire rearing period (days 1–49) showed significantly lower feed consumption in group I (2.57 kg/kg) than in groups III–V (*p* < 0.05). The use of different protein sources in the duck diets did not have a significant effect on feed consumption (FI) or BWG and FCR measurements taken in the second rearing period (i.e., on days 29–49 (*p* > 0.05)).

### 3.3. Meat Quality

Significantly higher (*p* < 0.05) weight of carcass was found in group II, which was fed on RSM and yellow lupin. A lower weight of carcass was measured in ducks from group IV, which was fed a diet of yellow lupin and pea. The weight of neck with skin was significantly higher (*p* < 0.05) in group II than in group V. The proportion of wings in the carcass of birds from group V (RSM, yellow lupin, narrow-leaved lupin and pea) was significantly higher (*p* < 0.05) than in group III. Other meat traits did not differ significantly (*p* > 0.05) between groups (Table 5). The content of muscles in duck carcasses was comparable in all groups. The weight of skin with subcutaneous fat was significantly higher (*p* < 0.05) in group II than in group IV. A significantly higher (*p* < 0.05) content of fat in carcass was also found in group II (yellow lupin and RSM) than in group IV (yellow lupin and pea) No significant differences in the weight of breast and leg muscles and their proportion in carcass were found. In every group, the proportion of total muscles was greater than 30% (Table 6). The analysis of physicochemical parameters of breast muscles showed significant differences (*p* < 0.05) in pH_15_. Birds fed with yellow and narrow-leaved lupins were characterized by a lower pH_15_ (5.78) than the other groups (>6.00). Breast muscles from group II were characterized by higher values of lightness (L*) but lower redness (a*) than in groups I and III. There were no significant differences (*p* > 0.05) between groups in water-holding capacity and drip loss. The content of protein in breast muscles was significantly higher (*p* < 0.05) in groups I and II than in other groups. The content of salt was significantly higher (*p* < 0.05) in groups I and V than in group II. The content of fat was significantly higher (*p* < 0.05) in groups I and III than in IV. A significantly higher (*p* < 0.05) content of water in breast muscles was found in groups II and IV (Table 7). The elasticity of breast muscles and strength of bones expressed as the maximum load applied to the tested carcass element and the compressive strength at the maximum load did not differ significantly, *p* > 0.05 (Table 8).

## 4. Discussion

Pea seeds are a good source of protein and starch, as are some varieties of lupin seeds [20,21,22]. Analyses of the chemical composition of legume seeds by other authors were also evaluated. Hejdysz et al. [23] showed that the CP level in narrow-leaved (257 g/kg DM) and yellow (416 g/kg DM) lupins was lower than in our own research (yellow lupin: 303 g/kg DM; narrow-leaved lupin: 424.7 g/kg DM). Rutkowski et al. [24] also used yellow lupin (cv. Mister) for laying-hen nutrition. The CP content was the lowest compared to our research (389.8 g/kg DM). In our study, the lupin seeds that were used had no starch. Nevertheless, Hejdysz et al. [23] noticed 10.2–10.3 g/kg of starch in lupin seeds. The total alkaloids in lupin seeds in duck diets were lower than those in the studies by Rutkowski et al. [24] and Hejdysz et al. [23]. Hejdysz et al. [25] used raw and extruded narrow-leaved lupin for broilers. Higher levels of CP, ADF, and NDF (37.8–38.1, 214.3–219.7, and 259.2–210.5 g/kg, respectively) were noticed compared with narrow-leaved lupin in our own research. Differences in chemical composition between studies were found because different varieties of lupins were used (cv. Boruta and cv. Sonet). In a study by Hejdysz et al. [26], the same varieties of lupins were used. A slightly smaller content of CP was found (41.6 g/100 g DM cv. Mister; 25.7 g/100 g DM cv. Sonet). The content of total oligosaccharides (RFO) in narrow-leaved lupin was slightly higher, and in yellow lupin, it was smaller than in our own studies. Raw pea seeds used in Hejdysz et al.’s [21] research on broiler chickens had a slightly higher content of CP (219 g/kg DM) than in our study (203.8 g/kg DM), as well as the extruded seeds (226 g/kg DM). Higher content of total RFO was noticed in both types of pea seeds, as well as the level of P-phyt. However, the concentration of RFO increased [10,27]. Diaz et al. [20] found that the concentration of CP in pea seeds was 212.5 g, whereas in extruded pea seeds, the CP level was 2.2 g lower. These studies also showed higher CP content than in our research. In the past, the CP level was highest in various pea seeds, from 236 to 266 g/kg DM [28]. Rutkowski et al. [29] used lupin seeds in laying-hen diets. The P-phyt. content in seeds was like the content in the seeds used in our study. In pea seeds, the P-phyt. content was almost two times greater (0.44%).

Legume seeds are a valuable protein-rich feed material, and they reportedly have a positive effect on performance parameters [30,31]. In the present study, the body weights of the ducks were higher than those in feeding trials by Jerabek et al. [32], who investigated the effects of a duck diet containing SBM and yellow lupin at levels of 50% and 100% of the feed ration. Olver [33,34] reported lower performance parameters in ducks fed a diet based on bitter lupin, which may be attributed to the negative effect of alkaloids. On the other hand, Mihok [35] found no lower preslaughter body weight in 7-week-old ducks fed a diet with 13–20% inclusion of lupin per feed ration. Rutkowski et al. [36] demonstrated that Pekin ducks fed on legume seeds for 8 weeks had about a 300 g higher body weight compared with ducks fed SBM. In the present study, the bodyweight of 7-week-old ducks fed a diet of legume seeds (groups II–IV) was 6–39 g higher compared with control birds. The abovementioned authors reported lower FCR in control groups. The present study did not confirm this as the FCR was higher in groups fed diets with legume seeds. The decrease in productivity parameters could be an effect of higher levels of NDF, P-phyt., raffinose, and antinutritive compounds in legumes [21,29].

A study by Kokoszyński et al. [37] revealed an 11.1–12.2% proportion of breast muscles and an 11.8–13.0% proportion of leg muscles in carcasses of 7-week-old ducks. On the other hand, the proportion of skin with subcutaneous fat in Pekin ducks reported by Bernacki and Adamski [38] and Mazanowski et al. [39] was in the range of 23.2–25.2%. Our study revealed a similar content of these elements in carcasses but a higher proportion of breast muscles (17.27–18.25%). The proportion of muscles in carcasses higher than 30% in each group and the low content of fat in groups II–IV suggest that the inclusion of legume seeds in the diet of fast-growing crossbreed meat ducks may be an effective solution. We found no significant differences between the groups in pH values, and reduced pH indicated normal glycolysis in the muscles after slaughter. The low pH value in group III could be the result of transport stress and using this group as the first for slaughtering. We think that the feeding factor did not have an influence on the pH value of the meat; however, excluding group III, no differences in pH_15_ and pH_24_ were found by Adamski et al. [40], who investigated the effects of different levels of dietary DGGS (source of protein) on meat traits in Pekin ducks. They also found no differences in the water-holding capacity of muscles. Higher color saturation (L*) and redness (a*) in ducks fed on yellow lupin and RSM may indicate a higher content of carotenoids per ration and a higher content of fat in carcasses, as shown in our study, where the content of fat was significantly higher (622.76 g). However, Witak et al. [41] found no effect of yellow lupin and RSM on the color of breast muscles. The present study revealed a 0.16–1.07% higher content of protein, a 0.58–1.44% higher content of water and a 0.29% higher content of fat compared with values reported by Kokoszyński et al. [42], apart from ducks fed a diet containing peas and yellow lupin, where the content of fat was 0.16% lower. The present study found a 0.30–0.45% higher content of protein compared with values measured by Kokoszyński et al. [42]. The content of water in breast muscles from ducks fed with the SBM diet was significantly the lowest, but it did not affect the water-holding capacity of muscles. The differences between the obtained results in the abovementioned and our studies could be due to the different origins of birds in each study (sex, age of birds, rearing season, etc.), the differences in the chemical composition of the seeds that were used in the diets and the types of plants in the diets. Duck performance and their carcass quality results are also dependent on the environmental conditions in the rearing house, which were not evaluated in the present study.

## 5. Conclusions

It can be concluded that the inclusion of various sources of vegetable protein alternatives to SBM in the diet of ducks for a 7 week rearing period provided the best results in birds fed concentrates containing yellow lupin and RSM (ratio of 1:0.31 in starter and 1:0.81 in grower). The growth performance parameters of these ducks were comparable to those achieved in birds fed an SBM diet. The values of meat traits were also comparable, which justifies the inclusion of lupin and RSM as an alternative to SBM. The higher content of fat in the carcasses can be a positive trait, considering the role of fat as a flavor carrier. Other tested concentrates may also partly replace SBM.

## Figures and Tables

**Table 1:** Chemical composition of legume seeds (in dry matter).

Contents	Pea (Tarachalska)	Yellow Lupin (Mister)	Narrow-Leaved Lupin (Sonet)
(%)			
Crude protein	20.38	42.47	30.3
ADF ^2^	9.09	19.39	20.46
NDF ^2^	16.5	25.16	25.5
Crude fat	1.03	3.52	4.87
Starch	59.01	-	-
Amino acids, % protein			
Aspartic Acid	12.23	11.21	9.98
Threonine	3.5	3.66	3.41
Serine	4.53	5.51	4.94
Glutamic Acid	15.77	25.22	19.54
Proline	3.8	3.61	3.71
Cysteine	0.86	1.91	0.82
Methionine	0.57	0.53	0.36
Glycine	4.11	4.28	4.05
Alanine	4.11	3.54	3.37
Valine	4.85	4.04	3.92
Isoleucine	3.94	4.3	4.01
Leucine	7.06	8.85	6.89
Tyrosine	2.83	3.09	3.24
Phenylalanine	4.59	4.42	4.02
Histidine	2.73	3.05	2.69
Lysine	7.91	6.66	5.45
Arginine	12.23	15.11	11.64
Antinutrients			
Total alkaloids (g/kg)	-	0.00085	0.0017
Oligosaccharides (g/kg)	62.91	134.27	58.06
Raffinose (g/kg)	8.36	10.21	7.07
Stachyose (g/kg)	27.13	82.8	37.23
Verbascose (g/kg)	27.42	41.26	13.76
P-phyt. ^2^ (%)	0.24	0.74	0.48
Tannin (g/kg)	0.021	-	-

^2^ ADF—acid detergent fiber; NDF—neutral detergent fiber; P-phyt.—phytic phosphorus.

**Table 2:** Concentrates used in the first phase of duck rearing.

Components (g/100 g)	Groups				
	Control	Experimental			
	I	II	III	IV	V
SBM ^2^	60.00	-	-	-	-
RSM ^2^	23.15	17.87	-	-	17.78
Yellow lupin	-	57.78	60.58	54.92	27.42
Narrow-leaved lupin	-	-	15.80	-	13.33
Pea	-	-	-	18.89	11.11
Potato protein	-	8.89	8.89	13.33	15.56
Soybean oil	9.38	7.60	6.44	4.44	6.67
Monocalcium phosphate	1.96	2.33	2.58	2.62	2.44
Fodder chalk	3.00	2.91	3.04	3.22	3.22
NaHCO_3_	0.58	0.58	0.58	0.58	0.58
DL-Methionine	0.38	0.49	0.58	0.49	0.38
Fodder salt	0.44	0.44	0.40	0.40	0.40
Premix ^1^	1.11	1.11	1.11	1.11	1.11
Calculated nutritional value (%)					
ME ^2^ (MJ/kg)	10.70	10.72	10.71	10.74	10.73
Crude protein	34.91	34.98	34.91	34.96	34.96
Lysine	1.90	1.90	1.87	1.87	1.90
Methionine	0.90	0.90	0.90	0.90	0.90
Threonine	1.42	1.38	1.38	1.40	1.40
Calcium	2.23	2.18	2.18	2.20	2.16
Phosphorus	0.58	0.58	0.58	0.58	0.58
Natrium	0.34	0.36	0.34	0.34	0.34
Chlorine	0.33	0.33	0.31	0.31	0.33
Components in diets (%)					
Concentrate	45				
Maize	25				
Triticale	30				
Calculated nutritional value of experimental diets ^2^ (%)					
ME ^2^ (MJ/kg)	11.91	11.92	11.92	11.93	11.92
Crude protein	20.94	20.97	20.94	20.96	20.96
Lysine	1.03	1.03	1.02	1.02	1.03
Methionine	0.50	0.50	0.50	0.50	0.50
Threonine	0.82	0.80	0.80	0.81	0.81
Calcium	1.03	1.01	1.01	1.02	1.01
Phosphorus	0.40	0.40	0.40	0.40	0.40
Natrium	0.16	0.17	0.16	0.16	0.16
Chlorine	0.19	0.19	0.18	0.18	0.19

^1^ Provides per kg diet: IU: vit. A 11 250, cholecalciferol 2500; mg: vit. E 80, menadione 2.50, vit. B12 0.02, folic acid 1.17, choline 379, d-pantothenic acid 12.5, riboflavin 7.0, niacin 41.67, thiamine 2.17, d-biotin 0.18, pyridoxine 4.0, ethoxyquin 0.09, Mn 73, Zn 55, Fe 45, Cu 20, I 0.62, Se 0.3. ^2^ SBM—soybean meal, RSM—rapeseed meal, ME—metabolic energy (MJ/kg).

**Table 3:** Concentrates used in the second phase of duck rearing.

Components (g/100 g)	Groups					
	Control		Experimental			
	I		II	III	IV	V
Triticale	17.44		4.76	1.07	-	-
SBM ^2^	38.89		-	-	-	-
RSM ^2^	27.05		34.49	-	-	24.89
Yellow lupin	-		42.22	41.13	56.49	41.00
Narrow-leaved lupin	-		-	40.00	-	8.89
Pea	-		-	-	26.29	7.33
Potato protein	-		-	-	2.22	-
Soybean oil	10.22		12.00	10.44	7.56	11.11
Monocalcium phosphate	1.00		1.22	1.60	1.60	1.33
Fodder chalk	3.07		2.89	3.16	3.31	2.98
NaHCO_3_	0.60		0.58	0.58	0.58	0.58
DL-Methionine	0.22		0.33	0.49	0.44	0.38
Fodder salt	0.40		0.40	0.42	0.40	0.40
Premix ^1^	1.11		1.11	1.11	1.11	1.11
Calculated nutritional value (%)						
ME ^2^ (MJ/kg)	11.48		11.53	11.48	11.51	11.45
Crude protein	33.67		33.53	33.07	33.64	33.53
Lysine	1.63		1.63	1.58	1.65	1.65
Methionine	0.78		0.78	0.78	0.78	0.78
Threonine	1.20		1.20	1.18	1.20	1.20
Calcium	2.09		2.09	2.06	2.06	2.06
Phosphorus	0.62		0.62	0.62	0.62	0.62
Natrium	0.35		0.35	0.35	0.35	0.35
Chlorine	0.36		0.34	0.34	0.34	0.34
Components in diets (%)						
Concentrate		45				
Triticale		55				
Calculated nutritional value of experimental diets (%)						
ME ^2^ (MJ/kg)	11.93		11.95	11.93	11.94	11.91
Crude protein	18.03		17.97	17.76	18.02	17.97
Lysine	0.85		0.85	0.83	0.86	0.86
Methionine	0.40		0.40	0.40	0.40	0.40
Threonine	0.64		0.64	0.63	0.64	0.64
Calcium	0.96		0.96	0.95	0.95	0.95
Phosphorus	0.35		0.35	0.35	0.35	0.35
Natrium	0.16		0.16	0.16	0.16	0.16
Chlorine	0.19		0.18	0.18	0.18	0.18

^1^ Provides per kg diet: IU: vit. A 11 250, cholecalciferol 2500; mg: vit. E 80, menadione 2.50, vit. B12 0.02, folic acid 1.17, choline 379, d-pantothenic acid 12.5, riboflavin 7.0, niacin 41.67, thiamine 2.17, d-biotin 0.18, pyridoxine 4.0, ethoxyquin 0.09, Mn 73, Zn 55, Fe 45, Cu 20, I 0.62, Se 0.3; ^2^ SBM—soybean meal, RSM—rapeseed meal, ME—metabolic energy (MJ/kg).

**Table 4:** Growth performance of ducks during the rearing period.

Group ^1^	BWG (g)			FI (g)			FCR (kg/kg)		
	Days 1–28	Days 29–49	Days 1–49	Days 1–28	Days 29–49	Days 1–49	Days 1–28	Days 29–49	Days 1–49
I	1732 ^a^	1449	3182 ^a^	3537	4639	8176	2.04 ^c^	3.21	2.57 ^b^
II	1720 ^ab^	1441	3161 ^a^	3576	4725	8302	2.08 ^bc^	3.29	2.63 ^ab^
III	1662 ^cd^	1448	3111 ^ab^	3543	4742	8285	2.13 ^ab^	3.29	2.67 ^a^
IV	1675 ^bc^	1437	3112 ^ab^	3546	4749	8296	2.12 ^b^	3.31	2.67 ^a^
V	1618 ^d^	1427	3046 ^b^	3526	4583	8109	2.18 ^a^	3.21	2.66 ^a^
SEM	10	11	14	8	30	36	0.01	0.03	0.01
*p*	0.001	NS	0.013	NS	NS	NS	<0.0001	NS	0.023

^a–d^—mean values marked in columns with different letters are significantly different between groups, *p* < 0.05, NS—no significance; BWG—body weight gain, FI—feed intake, FCR—feed conversion ratio; ^1^ I—soybean meal; II—yellow lupin and rapeseed meal (RSM); III—yellow lupin and narrow-leaved lupin; IV—pea and yellow lupin; V—RSM, yellow lupin, narrow-leaved lupin, and pea.

**Table 5:** Meat traits of 7-week-old ducks.

Group ^1^	Preslaughter Body Weight (g)	Weight of Carcass (g)	Dressing Percentage (%)	Weight and Proportion in Carcass				Weight of Offal (g)	Carcass Remains (g)
				Neck with Skin		Wings			
				g	%	g	%		
I	3347.50	2248.41	67.32	299.17	7.23	285.41	12.74	188.73	544.33
II	3361.00	2353.06 ^a^	70.01	317.32 ^a^	6.74	286.86	12.19	186.68	569.82
III	3260.00	2315.72	71.01	307.40	7.03	273.87	11.87 ^b^	195.86	602.00
IV	3149.00	2132.99 ^b^	67.74	284.88	7.35	274.00	12.85	170.11	562.78
V	3134.00	2187.73	69.80	278.08 ^b^	6.98	289.11	13.23 ^a^	185.92	532.34
SEM	25.69	21.68	0.46	3.89	0.13	2.32	0.12	2.76	14.17
*p*	NS	0.019	NS	0.020	NS	NS	0.006	NS	NS

^a^ and ^b^—mean values marked in columns with different letters are significantly different between groups, *p* < 0.05, NS—no significance; ^1^ I—soybean meal; II—yellow lupin and rapeseed meal (RSM); III—yellow lupin and narrow-leaved lupin; IV—pea and yellow lupin; V—RSM, yellow lupin, narrow-leaved lupin, and pea.

**Table 6:** Content of muscles and fat in 7-week-old ducks.

Group ^1^	Weight and Proportion in Carcass											
	Muscles						Skin with Subcutaneous Fat		Abdominal Fat		Total Fat	
	Breast		Legs		Total							
	g	%	g	%	g	%	g	%	g	%	g	%
I	410.82	18.25	291.56	13.00	702.38	31.25	530.37	23.73	23.60	1.06	553.97	24.79
II	413.17	17.52	301.60	12.79	714.77	30.31	598.89 ^a^	25.49	23.87	1.02	622.76 ^a^	26.51
III	398.60	17.27	305.24	13.21	703.84	30.48	549.77	23.71	23.85	1.03	573.62	24.74
IV	379.98	17.78	261.30	12.31	641.28	30.09	480.28 ^b^	22.50	17.93	0.84	498.21 ^b^	23.34
V	397.94	18.17	298.73	13.63	696.67	31.80	495.69	22.71	20.77	0.95	516.46	23.66
SEM	7.37	0.28	7.67	0.32	12.35	0.47	8.77	0.48	1.05	0.05	12.29	0.51
*p*	NS	NS	NS	NS	NS	NS	0.020	NS	NS	NS	0.022	NS

^a^ and ^b^—mean values marked in columns with different letters are significantly different between groups, *p* < 0.05, NS—no significance; ^1^ I—soybean meal; II—yellow lupin and rapeseed meal (RSM); III—yellow lupin and narrow-leaved lupin; IV—pea and yellow lupin; V—RSM, yellow lupin, narrow-leaved lupin, and pea.

**Table 7:** Carcass pH and physicochemical parameters of breast muscles in meat ducks.

Group ^1^	pH_15_	pH_24_	Colour ^2^			Water-Holding Capacity (%)	Drip Loss (%)	Protein (%)	Collagen (%)	Salt (%)	Fat (%)	Water (%)
			L*	a*	b*							
I	6.07	5.51	36.02 ^b^	17.24 ^a^	2.37	37.84	1.59	23.07 ^a^	1.43	0.10 ^a^	2.24 ^a^	75.88 ^b^
II	6.19	5.40	44.02 ^a^	12.19 ^b^	1.16	38.51	2.32	23.16 ^a^	1.40	0.06 ^b^	2.18	76.47 ^a^
III	5.78	5.50	36.60 ^b^	17.39 ^a^	2.40	40.01	0.15	22.76 ^b^	1.55	0.06	2.29 ^a^	76.20 ^b^
IV	6.27	5.45	40.08	15.11	2.83	41.34	1.65	22.75 ^b^	1.51	0.02	1.84 ^b^	76.74 ^a^
V	6.24	5.39	41.79	13.27	4.25	42.28	6.62	22.58 ^b^	1.48	0.10 ^a^	2.12	76.14 ^b^
SEM	0.03	0.08	71.55	0.54	0.31	0.82	1.22	0.04	0.04	0.02	0.04	0.07
*p*	NS	NS	<0.01	<0.05	NS	NS	NS	<0.05	NS	<0.05	<0.05	<0.05

^a^ and ^b^—mean values marked in columns with different letters are significantly different between groups, *p* < 0.05, NS—no significance; ^1^ I—soybean meal; II—yellow lupin and rapeseed meal (RSM); III—yellow lupin and narrow-leaved lupin; IV—pea and yellow lupin; V—RSM, yellow lupin, narrow-leaved lupin, and pea. ^2^ L*—lightness, a*—redness, b*—yellowness.

**Table 8:** Elasticity of breast muscles (N) and strength of femoral bones (N) in 7-week-old ducks *.

Group ^1^	Breast Muscles		Femur	
	Maximum Load Capacity (N)	Compressive Strength at Maximum Load (Mpa)	Maximum Load Capacity (N)	Compressive Strength at Maximum Load (Mpa)
I	96.93	0.24	95.77	0.24
II	96.68	0.24	96.85	0.24
III	98.18	0.24	95.60	0.24
IV	95.04	0.24	96.08	0.24
V	96.12	0.24	96.54	0.24
SEM	0.58	0.00	0.34	0.00
*p*	NS	NS	NS	NS

* no significant differences, *p* > 0.05, NS—no significance; ^1^ I—soybean meal; II—yellow lupin and rapeseed meal (RSM); III—yellow lupin and narrow-leaved lupin; IV—pea and yellow lupin; V—RSM, yellow lupin, narrow-leaved lupin, and pea.
